# A New Case of *PITX1*-Related Mandibular–Pelvic–Patellar (MPP) Syndrome

**DOI:** 10.3390/clinpract16020031

**Published:** 2026-01-29

**Authors:** Evgeniya Melnik, Ekaterina Petrova, Tatiana Markova, Ksenya Zabudskaya, Elena Dadali

**Affiliations:** 1 Research Centre for Medical Genetics, Moskvorechye St., 1, 115522 Moscow, Russia; markova@med-gen.ru (T.M.); genclinic@yandex.ru (E.D.); 2 H. Turner National Medical Research Center for Children’s Orthopedics and Trauma Surgery of the Ministry of Health of the Russian Federation, Parkovaya 64–68, Pushkin, 196603 Saint Petersburg, Russia

**Keywords:** *PITX1*, Mandibular–Pelvic–Patellar syndrome, MPP, patellar aplasia, arthrogryposis, congenital lower limb deformities

## Abstract

**Background**: The *PITX1* gene encodes a transcription factor that plays a crucial role in the development of the lower limbs, pelvis, and structures derived from the first branchial arch. Pathogenic variants in *PITX1* are associated with a limited spectrum of rare disorders, including congenital talipes equinovarus with or without long bone anomalies and/or mirror-image polydactyly, and Liebenberg syndrome. In 2020, a novel clinical phenotype, Mandibular–Pelvic–Patellar (MPP) syndrome, resulting *PITX1* missense variants, was proposed. **Case presentation**: We report the fourth documented case of MPP syndrome worldwide, identified in a 17-year-old female patient presenting with congenital lower limb deformities, patellar aplasia, and micrognathia. Whole-genome sequencing revealed a heterozygous *PITX1* missense variant NM_002653.5: c.412A>C, p.(Lys138Gln). The clinical phenotype included knee flexion contractures and severe equinovarus and planovalgus foot deformities requiring multiple staged reconstructive surgical procedures. **Conclusions**: This case supports recognition of MPP syndrome as a clinically and genetically distinct *PITX1*-related disorder. Our findings expand the phenotypic spectrum of MPP syndrome and suggest that severe congenital foot deformities represent a consistent and clinically relevant feature of this condition.

## 1. Introduction

Limb development during embryogenesis is tightly regulated by a complex interplay of transcription factors and signaling pathways. Among these, homeobox genes play a critical role in regional patterning and organogenesis [1,2,3]. The *PITX1* gene, located on chromosome 5q31.1, encodes a highly conserved transcription factor essential for hindlimb specification and morphogenesis [1]. In humans, *PITX1* is predominantly expressed in lower extremities, pelvis, first pharyngeal arch, and pituitary gland, and its dysfunction leads to a broad spectrum of limb anomalies.

To date, two *PITX1*-associated disorders have been recognized in the OMIM database. The first includes congenital clubfoot with or without long-bone abnormalities and/or mirror polydactyly (OMIM #119800), initially described by Gurnett et al. (2008), who identified a missense variant affecting the homeodomain and suggested a dominant-negative disease mechanism [4]. This phenotype is characterized by marked clinical variability and incomplete penetrance, with manifestations ranging from isolated bilateral clubfoot to complex malformations. The second *PITX1*-related phenotype is Liebenberg syndrome (OMIM #186550), characterized by upper limb malformations resembling hindlimb morphology due to ectopic PITX1 expression caused by chromosomal rearrangements [5,6].

In 2020, Morel et al. expanded the *PITX1* phenotype spectrum by describing three patients with a distinct constellation of mandibular hypoplasia (microretrognathia), characteristic pelvic anomalies, patellar hypo-/aplasia with knee contractures, and genital anomalies in males, associated with two *PITX1* missense variants. The authors proposed this disorder as “Mandibular–Pelvic–Patellar (MPP) syndrome” [7]. However, the number of reported cases remains extremely limited.

Herein, we present the fourth known case of MPP syndrome worldwide, associated with the *PITX1* missense variant c.412A>C, p.(Lys138Gln). This report contributes to the expanding phenotypic characterization and strengthens the evidence supporting MPP syndrome as a clinically and genetically distinct *PITX1*-related disorder.

## 2. Case Presentation

The proband was a 17-year-old girl who presented with lower limb deformities and gait disturbance. She was the first child of healthy, non-consanguineous Russian parents. Pregnancy was uneventful, and delivery occurred at 39 weeks of gestation with a birth weight of 3838 g (+0.9 SD), length of 46 cm (−1.55 SD). From birth she exhibited muscular hypotonia, equinovarus deformity of the right foot, equino–plano–valgus deformity of the left foot, and flexion contractures of the knees. Based on the combination of orthopedic abnormalities, a diagnosis of arthrogryposis of the lower limbs was suspected. Initial management consisted of serial casting. Cognitive and speech development were normal. Motor development was delayed due to lower limb orthopedic abnormalities. With the goal of correcting severe deformities and enabling independent mobility, the patient received comprehensive, staged orthopedic surgery since the age of 6. The surgical management comprised corrective femoral osteotomies, stabilizing arthrodesis of the foot joints, muscle-balancing tendon transfers, and temporary hemiepiphysiodesis for guided growth modulation. This multistage surgical strategy ultimately allowed the patient to achieve independent ambulation by age 10, which she maintains at the age of 17.

**Figure 1:**
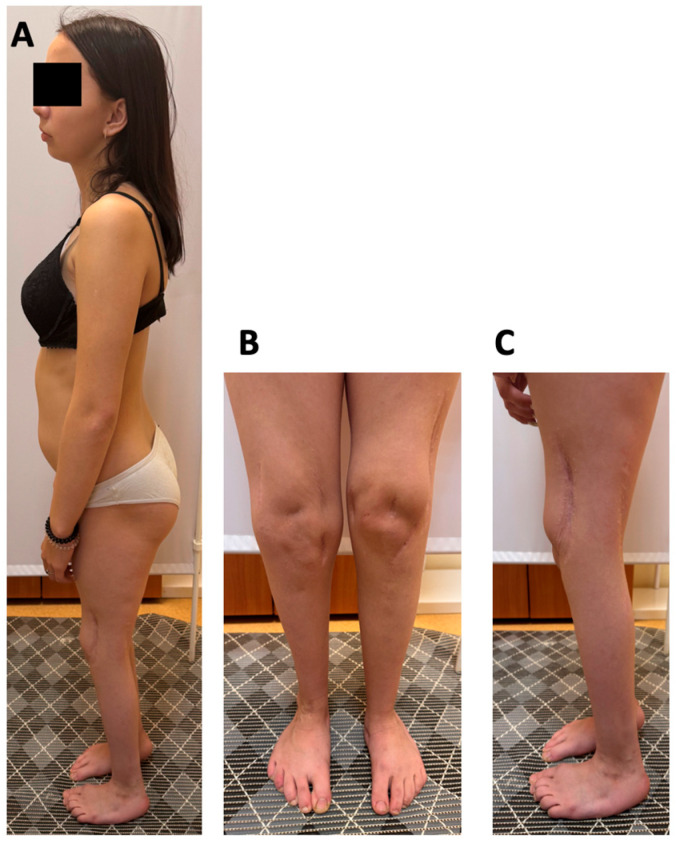
Clinical features of the proband. (**A**) Microretrognathia, low-set ears. (**B**,**C**) Muscle hypotrophy of the thighs and calves, shortened toes, knee flexion contractures, equinovarus deformity of the right foot and equino–plano–valgus deformity of the left foot.

At 17 years of age, the proband’s weight was 44.5 kg (−1.52 SD) and her height 149 cm (−2.09 SD). Physical examination revealed microretrognathia, low-set ears, 2-cm shortening of the left leg, restricted knee flexion (70–80°), absence of patellar on palpation, decreased tendon reflexes in the lower limbs, muscle hypotrophy of the thighs and calves, and shortened toes. The right foot demonstrated a rigid equinovarus deformity at 120° with absent ankle mobility, while the left foot was plantigrade with mild limitation of motion (dorsiflexion 10°, plantarflexion 20°) (Figure 1). She was able to walk independently, bearing weight on the forefoot of the right foot. Hip range of motion was preserved, and the upper extremities were unaffected.

**Figure 2:**
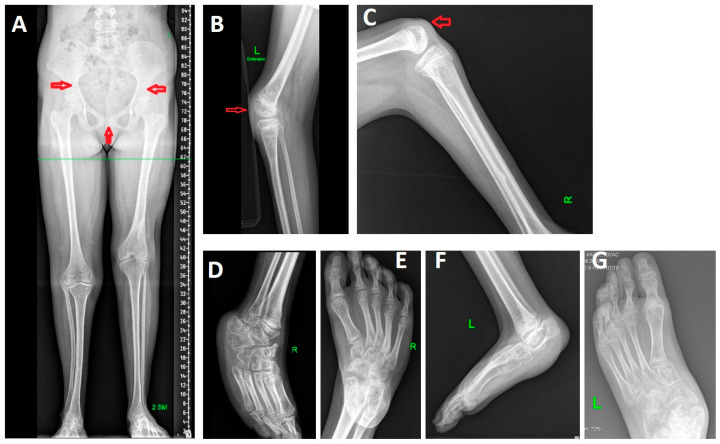
Radiographs of the proband’s lower limbs. (**A**) Panoramic anteroposterior radiograph of the lower limbs of a 17-year-old female patient. A shortening of the left lower limb by 2 cm is observed (4 cm in the right tibia and 6 cm in the left femur). The iliac bones exhibit an atypical shape, and the pubic symphysis is widened (indicated by red arrows). (**B**,**C**) Lateral radiographs of the knees of a 12-year-old female patient. Flexion contractures are present, measuring 70° on the right and 35° on the left. Bilateral patellar aplasia is evident (indicated by red arrows). (**D**–**G**) Anteroposterior and lateral radiographs of the feet of an 11-year-old female patient. The right foot demonstrates an equinovarus deformity (**D**,**E**), while the left foot shows an equino–plano–valgus deformity (**F**,**G**).

Radiographs of the lower limbs revealed 2-cm shortening of the left leg, atypical morphology of the iliac bones, a wide pubic symphysis, bilateral patellar aplasia, flexion contractures of the knees, foot deformities (Figure 2).

Given the presence of contractures and patellar aplasia, nail–patella syndrome (OMIM #161200) was initially considered. However, Sanger sequencing of the entire coding sequence and exon–intron boundaries of the *LMX1B* gene revealed no likely pathogenic or pathogenic variants.

Whole-genome sequencing subsequently identified a heterozygous nucleotide variant in exon 3 of *PITX1* (chr5:135029312T>G (hg38)), predicting the amino acid substitution NM_002653.5 (MANE Select transcript): c.412A>C, p.(Lys138Gln). This variant previously reported as pathogenic and altering *PITX1* transactivation ability in a single publication in a patient with MPP [7].

**Figure 3:**
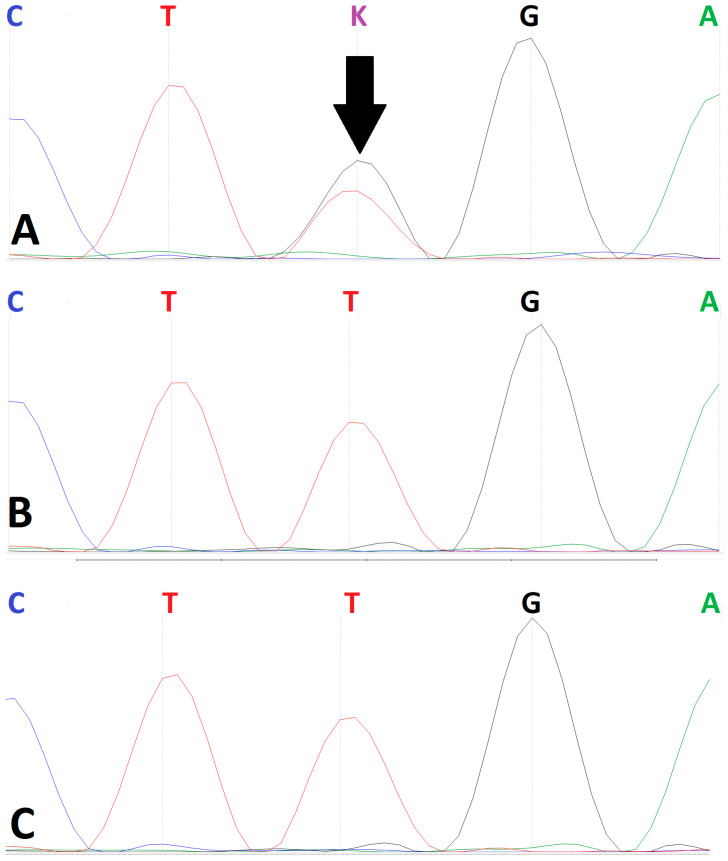
Family segregation analysis of the *PITX1* variant. The proband (arrow) (**A**) is heterozygous for the *PITX1* (NM_002653.5): c.412A>C, p.(Lys138Gln) variant. The proband’s maternal aunt (**B**) and maternal grandmother (**C**) tested negative for the variant. Parental samples were unavailable.

Sanger sequencing confirmed the heterozygous state of the c.412A>C variant in the proband (Figure 3). Direct confirmation of a *de novo* origin was precluded by the unavailability of parental biosamples. However, analysis of available maternal-line relatives (the proband’s maternal aunt and grandmother) failed to detect the variant. This finding indicates that the variant was not inherited from the maternal lineage, though this cannot be definitively confirmed.

The variant was interpreted according to the ACMG guidelines. The formal classification as likely pathogenic was supported by the following evidence criteria:

- PM2 (absent from, or at extremely low frequency in, population databases). The variant is absent from gnomAD v3.1.2 population frequency database; in gnomAD v4.1.0 population frequency database only 1 heterozygous allele out of 1,595,294 was registered (frequency 0.00006268%—does not exceed the recommended value for autosomal dominant diseases of 0.01%) (http://gnomad.broadinstitute.org/, accessed on 21 February 2025).
- PP3 (computational analyses across multiple platforms suggest a deleterious effect). In silico prediction tools (FATHMM, fathmm_MKL_coding, M_CAP, PROVEAN, SIFT4G, SIFT, Polyphen2_HVAR, Polyphen2_HDIV, DEOGEN2, LRT, PrimateAI) consistently predict the variant to be damaging. Comprehensive pathogenicity assessment using meta-predictors: MetaSVM—pathogenic; MetaLR—pathogenic.
- PS3 (functional evidence from established in vitro or in vivo studies supports a damaging effect) [7].
- PP2 (missense variant in a gene that has a low rate of benign missense variation and where missense variants are a common mechanism of disease). In HGMD^®^ Professional v. 2025.1 and ClinVar databases missense variants are reported as pathogenic.
- PP5 (reputable source recently reports variant as pathogenic, but the evidence is not available to the laboratory for independent evaluation). Applied based on the single prior report classifying it as pathogenic [7].

The combination of PM2, PP3, PS3, PP2 and PP5 supports the final classification of likely pathogenic.

Based on the molecular findings in conjunction with the clinical and radiographic phenotype, the diagnosis of MPP syndrome was established. At the time of writing (December 2025), this syndrome has not yet been annotated in OMIM.

## 3. Discussion

We report the fourth confirmed case of MPP syndrome and the second independent observation of the *PITX1* variant c.412A>C, p.(Lys138Gln). Together with the previously published data, our case firmly supports recognition of MPP syndrome as a distinct nosological entity within the *PITX1*-related disorder spectrum. The phenotype in our patient demonstrates striking overlap with previously described cases, confirming the diagnostic triad initially proposed for MPP syndrome: mandibular hypoplasia, characteristic pelvic dysmorphology with a wide pubic symphysis, and patellar aplasia or hypoplasia accompanied by knee contractures. In our case, we identified the NM_002653.5: c.412A>C, p.(Lys138Gln) variant, previously reported in a patient with Pierre Robin sequence, micropenis, cryptorchidism, and severe knee flexion contractures [7]. Importantly, our observation further expands the clinical spectrum by demonstrating that severe congenital foot deformities requiring extensive orthopedic management represent a consistent and clinically significant component of the syndrome rather than an occasional finding. This emphasizes that MPP syndrome is not limited to the pelvis and knees but involves a broader lower limb developmental disturbance.

At the molecular level, the c.412A>C (p.Lys138Gln) variant is located within the DNA-binding domain of *PITX1*. Functional studies demonstrated profound impairment of transcriptional activity, with a nearly sevenfold reduction in transactivation capacity compared with wild-type protein, supporting a loss-of-function or dominant-negative effect [7]. Although earlier *PITX1* missense variants associated with congenital clubfoot also affect the homeodomain, the clinical phenotype differs substantially [4]. The coexistence of distinct phenotypic outcomes resulting from mutations in the same functional region may reflect differential residual protein activity, variable transcriptional effects on downstream targets, or tissue-specific sensitivity to PITX1 deficiency. Furthermore, the second MPP-associated variant, p.(Gly265Cys), located outside the homeodomain, suggests that pathogenic alterations in multiple functional regions of PITX1 may converge toward the MPP phenotype, complicating genotype–phenotype prediction [7].

To date, only six single-nucleotide variants in *PITX1* have been reported to cause lower limb malformations. In addition to three missense variants identified in patients with congenital clubfoot, with or without anomalies of long bones and/or mirror-image polydactyly, and in those with MPP syndrome, further variants have been described in patients with isolated congenital clubfoot: c.131A>G, p.(Glu44Gly) [8], c.169G>T, p.(Glu57Ter) [9], and c.292delA, p.(Ser98Alafs7) [10]. Moreover, Alvarado et al. described a 241-kb microdeletion at chromosome 5q31.1, encompassing *PITX1*, in a three-generation family with isolated foot deformities [11]. In 2012, Klopocki et al. reported an intragenic deletion in *PITX1* (c.765_799del, p.(Ala256Argfs303)) in a fetus presenting with bilateral preaxial polydactyly, clubfoot, and right-sided tibial hemimelia. Additionally, deletions of 4.9 Mb and 5.7 Mb encompassing *PITX1* were identified in two fetuses with clubfoot and mirror polydactyly [12]. One fetus lacked a bone of the left lower leg, while the other exhibited popliteal pterygium and cleft palate. Collectively, these findings underscore the broad spectrum of lower limb and pelvic malformations associated with *PITX1* dysfunction, ranging from isolated congenital foot deformities to complex syndromic presentations. This highlights the need for wider implementation of molecular genetic approaches, including next-generation sequencing and chromosomal microarray analysis, in the etiological investigation of patients with congenital lower limb anomalies of varying severity [13].

From a clinical perspective, our case highlights two important diagnostic considerations. First, MPP syndrome should be included in the differential diagnosis of patients with combined mandibular hypoplasia, pelvic dysplasia, patellar anomalies, and congenital deformities of the lower extremities. Second, *PITX1* sequencing should be incorporated into molecular diagnostic pipelines for such patients, enabling distinction from clinically overlapping entities such as LMX1B-related nail–patella syndrome. Importantly, despite severe musculoskeletal involvement, our patient achieved independent ambulation following staged orthopedic management, indicating that functional outcomes can be substantially improved with timely and tailored surgical care.

Further accumulation of clinical observations and functional studies is required to better delineate the full phenotypic spectrum, clarify disease mechanisms, and develop evidence-based management recommendations for this rare but clinically impactful syndrome.

## 4. Material and Methods

### 4.1. Literature Search Strategy

To verify the rarity of the condition, we conducted a structured literature search in PubMed, Scopus, and Google Scholar databases. The search was performed in December 2025 using the following keywords and combinations: “PITX1”, “Mandibular–Pelvic–Patellar syndrome”, “MPP syndrome”, “pelvic dysplasia”, “patellar aplasia”, “lower limb malformations”. No language restrictions were applied. Reference lists of identified publications were also screened. This search yielded only the previously reported three cases associated with *PITX1*-related MPP phenotype in one published article, confirming the extreme rarity of the condition.

### 4.2. Whole-Genome Sequencing

Whole-genome sequencing was conducted on the DNBSEQ-T7RS platform. For library preparation, genomic DNA was first extracted from whole blood using the manufacturer’s protocol for the MGIEasy Magnetic Beads Genomic DNA Extraction Kit (MGI, Shenzhen, China). DNA concentration was then precisely measured using a Qubit™ dsDNA HS Assay Kit (Thermo Fisher Scientific, Waltham, MA, USA).

Starting with 1000 ng of gDNA, libraries were constructed using the MGIEasy Fast PCR-FREE FS DNA Library Prep Set V2.0 (MGI, Shenhzen, China) per the manufacturer’s protocol. This included enzymatic fragmentation, bead-based cleanup, end-repair, and adapter ligation (MGIEasy UDB PF Adapters-96 Kit).

Library fragment size was verified using an Agilent 4200 TapeStation D1000 ScreenTape (Agilent, Santa Clara, CA, USA), and concentration was measured with a Qubit™ dsDNA Quantification Assay Kit.

Qualified libraries were circularized, amplified via rolling-circle replication to form DNA nanoballs, and loaded onto a flow cell with the DNBSEQ-T7RS High-throughput Sequencing Kit (MGI, Shenzhen, China). Sequencing was performed on the DNBSEQ-T7RS platform (MGI, Shenzhen, China) for paired-end 150 bp reads.

Raw sequencing reads were preprocessed by trimming adapter sequences and removing low-quality bases at read ends using cutadapt (v.4.2) with parameters “--trim-n --quality-cutoff 30,30 --error-rate 0.1 --times 99 --minimum-length 0 --pair-filter both --interleaved”. Alignment of paired-end reads to the GRCh38 genome assembly was performed with BWA-MEM (v0.7.17), using the parameters “-k 30 -K 100000000 -Y”. The SAM files were converted to sorted BAM format using SAMtools (v1.16.1).

Sequencing yielded 752,636,920 total reads, providing 36× average coverage. Raw read quality was assessed with FastQC (v0.11.9). After alignment, PCR duplicates were marked using GATK MarkDuplicatesSpark (v4.3.0.0).

All processing of genome sequencing data was conducted using the internal NGS-Data-Genome online service (Research Centre for Medical Genetics, Moscow, Russia; FIP registration number 2021662119). Within this pipeline, variants were called using the DeepVariant algorithm implemented in NVIDIA Clara Parabricks (v4.0.0) with key parameters “--disable-use-window-selector-model --normalize-reads --track-ref-reads --min-mapping-quality 10”. Called variants were subsequently annotated in accordance with the Human Genome Variation Society (HGVS) nomenclature (v21.1.3).

To assess pathogenicity, variant allele frequencies were annotated using population databases (1000 Genomes Project, gnomAD v3.1.2), and clinical relevance was determined through a review of the Online Mendelian Inheritance in Man (OMIM) database and the relevant scientific literature.

Pathogenicity classification was conducted in strict accordance with the American College of Medical Genetics and Genomics (ACMG) guidelines.

### 4.3. Segregation Analysis

To assess familial segregation, Sanger sequencing was conducted for the proband and available relative (maternal aunt and grandmother). Sequencing was carried out on an ABI Prism 3500 Genetic Analyzer (Applied Biosystems, Foster City, CA, USA) according to the manufacturer’s protocol. Primers were designed to amplify the relevant genomic region. The identified variant is described according to the MANE Select transcript NM_002653.5, following standard HGVS nomenclature guidelines.

## 5. Conclusions

Our findings support recognition of Mandibular–Pelvic–Patellar (MPP) syndrome as a clinically and genetically distinct syndrome within the spectrum of *PITX1*-related disorders. The clinical features observed in our case suggest that the phenotypic spectrum of this syndrome is broader than previously described. In particular, we propose that congenital foot deformities be regarded as a characteristic component, given their recurrence among affected individuals and the need for orthopedic interventions. Moreover, our experience indicates that staged orthopedic management may enable favorable functional outcomes despite severe congenital deformities. Continued accumulation of clinical observations will help refine the phenotypic variability, expand the spectrum of pathogenic *PITX1* variants and broaden understanding of genotype–phenotype correlations. This case also underscores the critical role of molecular genetic testing in the diagnostic evaluation of patients with congenital lower limb malformations.

## Data Availability

The original contributions presented in this study are included in the article. Further inquiries can be directed to the corresponding author.
